# Extract from the Speech of Gov. Wise, of Virginia

**Published:** 1860-03

**Authors:** 


*Extract from the Speech of Governor Wise, of Virginia.*

We take the following extract from the speech of Governor Wise to the Medical Students at Richmond, Virginia, on the occasion of the exodus from Philadelphia.

"Here in the midst of this metropolis, is erected an institution of Virginia's own, on the very spot where God has grown the snake-root to cure the ague and fever. [Laughter and applause.] Then we have a University, a Faculty, a hospital; and will you tell me Virginia's doctors cannot, at home, in Virginia, learn to cure her diseases as well as they can by spending millions in Philadelphia for learning there, how to cure disease? I was frequently sick in Philadelphia, and have had the doctors there waiting upon me. On one of these occasions I had no less a man than the illustrious Virginian, Nathan Chapman, waiting on me. Mr. Chapman was a most eloquent orator, and as eloquent in colloquy. On one occasion I got tired of his conversation, and I said to him, "Doctor, have you no patients to attend?" Says he, "Sir," in his nasal tone, "I have killed almost all, and cured the rest." [Laughter.] Said I, "Sir, have you no lectures to write? For God's sake let me alone!" Says he, "Sir, I wrote my lectures forty years ago, and I only have now to furbish them up with a few new anecdotes." [Laughter.] "Doctor," said I, "will that do in this enlighted age?" "Yes," said he, "it is good enough to make corn-crackers for Virginia." [Loud Laughter.] There is more philosophy in this anecdote than perhaps you may now likely give to it. Those high-pretending institutions, after they get their fame up, have more pretensions about them than there is of reality in them. The institution that is young; the institution that is rising; the institution that is to make a reputation, is the one where, if you are earnest and zealous in your search after science, you will derive most benefit. The school that is unknown and unsung to fame—there is the place to get the real waters of the true Pierian Spring."

